# Delta Radiomics Can Predict Distant Metastasis in Locally Advanced Rectal Cancer: The Challenge to Personalize the Cure

**DOI:** 10.3389/fonc.2020.595012

**Published:** 2020-12-03

**Authors:** Giuditta Chiloiro, Pablo Rodriguez-Carnero, Jacopo Lenkowicz, Calogero Casà, Carlotta Masciocchi, Luca Boldrini, Davide Cusumano, Nicola Dinapoli, Elisa Meldolesi, Davide Carano, Andrea Damiani, Brunella Barbaro, Riccardo Manfredi, Vincenzo Valentini, Maria Antonietta Gambacorta

**Affiliations:** ^1^Dipartimento Diagnostica per Immagini, Radioterapia oncologica ed Ematologia, Fondazione Policlinico Universitario Agostino Gemelli IRCCS, Rome, Italy; ^2^Radiology Department, La Princesa University Hospital, Madrid, Spain; ^3^Dipartimento Universitario di Scienze Radiologiche ed Ematologiche, Università Cattolica del Sacro Cuore, Rome, Italy

**Keywords:** radiomics, rectal cancer, predictive model, distant metastasis, neoadjuvant chemoradiotherapy

## Abstract

**Purpose:**

Distant metastases are currently the main cause of treatment failure in locally advanced rectal cancer (LARC) patients. The aim of this research is to investigate a correlation between the variation of radiomics features using pre- and post-neoadjuvant chemoradiation (nCRT) magnetic resonance imaging (MRI) with 2 years distant metastasis (2yDM) rate in LARC patients.

**Methods and Materials:**

Diagnostic pre- and post- nCRT MRI of LARC patients, treated in a single institution from May 2008 to June 2015 with an adequate follow-up time, were retrospectively collected. Gross tumor volumes (GTV) were contoured by an abdominal radiologist and blindly reviewed by a radiation oncologist expert in rectal cancer. The dataset was firstly randomly split into 90% training data, for features selection, and 10% testing data, for the validation. The final set of features after the selection was used to train 15 different classifiers using accuracy as target metric. The models’ performance was then assessed on the testing data and the best performing classifier was then selected, maximising the confusion matrix balanced accuracy (BA).

**Results:**

Data regarding 213 LARC patients (36% female, 64% male) were collected. Overall 2yDM was 17%. A total of 2,606 features extracted from the pre- and post- nCRT GTV were tested and 4 features were selected after features selection process. Among the 15 tested classifiers, logistic regression proved to be the best performing one with a testing set BA, sensitivity and specificity of 78.5%, 71.4% and 85.7%, respectively.

**Conclusions:**

This study supports a possible role of delta radiomics in predicting following occurrence of distant metastasis. Further studies including a consistent external validation are needed to confirm these results and allows to translate radiomics model in clinical practice. Future integration with clinical and molecular data will be mandatory to fully personalized treatment and follow-up approaches.

## Introduction

Colorectal cancer is the third most incident malignancy and the fourth in cancer-related death, being more prevalent in regions with high human developmental index (1).

The standard treatment of locally advanced rectal cancers (LARC) is neoadjuvant chemoradiotherapy (nCRT) followed by surgery with total mesorectal excision (TME) (2, 3).

The combination of nCRT and surgery has improved local control (LC) of the disease in LARC patients, but it does not affect the disease-free (DFS) and overall survivals (OS) (3).

Recurrence in the form of distant metastases (mainly affecting the liver) is the main cause of treatment failure and near 25% of treated LARC patients develop metastases in 5 years (4, 5). Early development of metastases (within 2 years) identifies biologically aggressive tumors and is considered a strong predictor of OS (3). Identification of patients with higher risk of developing distant metastasis within 2 years (2yDM) represents therefore a topic of great interest for the clinical community, as it could allow a more accurate personalized management, defining more strict clinical and imaging vigilance or even proposing more intensive treatments.

Mesorectal fascia involvement, depth of invasion, lymphovascular invasion and lymph node involvement currently represent key features that imply worse prognosis (6, 7).

Similarly, pathological response predicts patient prognosis and outcome, regarding local or distant recurrence and OS (3).

Rectal cancer is a rather heterogeneous disease, both inter and intratumoral, in space and time, regarding histology, immunochemistry and genetic profiles. This heterogeneity in the tumor cell populations may explain the variability of biological behavior and response to therapy existing in rectal cancer (8, 9). Tumor heterogeneity can be reflected in imaging, arising the opportunity of identifying imaging biomarkers that correlate with the tumor’s biological behavior (10).

Magnetic resonance imaging (MRI) is the standard imaging technique for local staging and re-evaluation after nCRT in rectal cancer (11), although it still has some limitations in the clinical and pathological prediction staging (12, 13).

In this context, radiomics, can play a key role, providing minable data from standard radiological images and exploring quantitative features which can describe tumor heterogeneity and other intrinsic characteristics that could correlate with its biological behavior (10, 14).

Several previous experiences have described radiomics based models for the prediction of pathological complete response (pCR) after nCRT (9, 15–20), clinical outcomes (8, 21) and grading in LARC (22, 23).

The already existing data show that active oncological treatments can modify radiomics features, an approach known as “delta radiomics”, and that the evaluation of these changes may successfully predict tumor behavior in terms of synchronous or metachronous distant metastasis (DM), DFS and OS (4, 14–16, 21, 24–26).

The purpose of this study is to assess the ability of the delta radiomics approach in predicting 2yDM in LARC, combining radiomics features extracted from staging and post-treatment MRI (24, 27).

## Materials and Methods

### Population of Study

The target population were LARC patients treated with nCRT and subsequently addressed to surgery.

We retrospectively and consecutively selected patients from our institution, a national reference centre for rectal cancer treatment, between May 2008 and June 2015, who met the following inclusion criteria: (a) patients older than 18 years old; (b) with pathologically proven rectal adenocarcinoma (including the mucinous variant that was regarded as separate); (c) clinical stage T3-4 N0, T1-4 N1-2 or with mesorectal fascia involvement (MRF+) according to the AJCC TNM 7^th^ edition; (d) nCRT followed by surgery at our centre; (e) with both pre-treatment (staging) and post-treatment (re-evaluation) MRI performed in our institution; (f) maximum intervals of 3 months between the end of nCRT and post-treatment MRI (14); (g) clinical and imaging follow-up of at least 3 years from surgery.

All patients underwent radiotherapy treatment with a prescribed total dose of 45 Gy (1.8 Gy/die) delivered on the whole mesorectum and the drainage nodal stations and a boost on the tumor plus corresponding mesorectum up to 55 Gy with simultaneous integrated boost (SIB) technique (2.2 Gy/die) or to 50.4 Gy in case of sequential boost.

The considered neoadjuvant chemotherapy regimens were: CapOx (60 mg/m^2^ of iv oxaliplatin at the first day plus 1300 mg/(die*m^2^) of oral capecitabine, day to 1^st^ to 7^th^, q7), capecitabine alone (1300 mg/m^2^ day 1^st^ to 7^th^ or 1^st^ to 5^th^ q7 during radiotherapy), or 5-fluorouracil (225 mg/(mq*die) from 1^st^ to 7^th^ day q7 during radiotherapy) depending on clinical stage and clinical patients compliance.

Surgery was performed at from 8 to 12 weeks from the end of nCRT and included: anterior resection (AR), abdominal-perineal resection (APR), transanal endoscopic microsurgery (TEM).

Adjuvant chemotherapy was administered for selected patients with clinical high-risk factors (i.e. cT4 and ypT3-4, ypN1-2, lymphovascular invasion of the tumor, TRG=4, etc.).

Adjuvant chemotherapy was based on 5-fluorouracil or capecitabine with or without oxaliplatin.

### MRI Protocol

All MRI images were acquired using 1.5 T scanners (Signa Excite, GE Medical Systems, Milwaukee, Wisconsin, USA), with a pelvic phased-array surface coil.

All patients were scanned in supine position. An enema of ultrasound gel (63 cm^3^) to distend rectal lumen and limit luminal air and 20 mg of intramuscular hyoscine-N-butylbromide (Buscopan; Boehringer Ingelheim Italia, Florence, Italy), as antiperistaltic, were administered to reduce artefacts. All MRI followed the standard protocol of our centre for rectal cancer (T2-weighted FSE images in axial, coronal and sagittal planes, T2-weighted FSE 3D high-resolution images perpendicular to the tumor, and axial DWI using b values of 0 and 1000 s/mm2) (12).

For radiomics analysis T2-weighted fast spin-echo 3D high-resolution images acquired in a plane orthogonal to the tumor longitudinal axis were used, according to the fact that it is the main staging modality and its use for radiomics was previously widely explored in rectal cancer (11–16, 25).

Pixel spacing of these images was not greater than 0.8 mm and slice thickness was not higher than 3 mm.

For each patient, pre and post nCRT MRI were analyzed.

MRI images were then uploaded on a radiotherapy delineation console (Eclipse, Varian Medical System™, Palo Alto, California, USA) for gross tumor volume (GTV) segmentation.

Gross tumor volumes (GTV) were delineated by an abdominal radiologist and blindly reviewed by a radiation oncologist (28).

Contouring and revision of MRI images were blinded with respect to all clinical data including the histology of the tumor, treatment received, surgical results and clinical evolution.

In case of disagreement between the two experts a final GTV was agreed on consensus.

Tumor response on MRI after nCRT was classified as “complete”, “partial” or “stable”. Complete response was considered when tumoral tissue had completely disappeared on the analyzed T2-weighted images, in absence of any suspicious residual tissue of intermediate signal or no residual hyperintense signal in DWI sequences (12, 13). In these cases of apparent complete response at MRI, the former tumor bed was contoured.

### Radiomic Analysis

**Figure 1:**
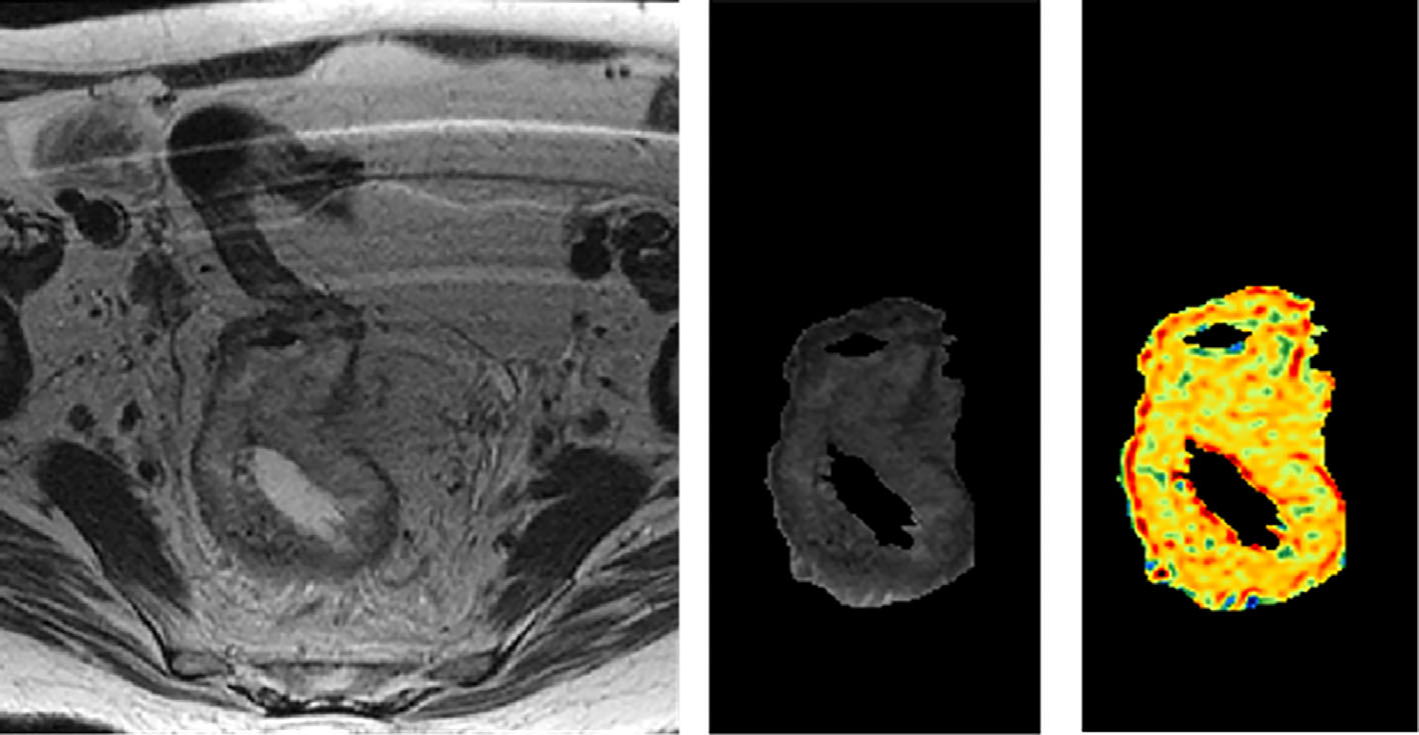
GTV extraction and filter application.

Radiomics features were extracted from both pre-nCRT and post-nCRT MR images using an in-house developed radiomics software, called Moddicom (29, 30). Different families of features were extracted: statistical, morphological, textural grey level co-occurrence matrix (GLCM), textural grey level run length matrix (GLRLM), textural grey level size zone matrix (GLSZM), and fractals. GTV extraction and filter application are shown in **Figure 1**.

Before extracting the statistical and textural radiomics features, a Laplacian of Gaussian (LOG) filter was applied to the MR images, considering 13 different sigma values in the range of 0.4–1.6 mm. Fractal features were calculated on the processed MR images, as described by Cusumano et al. (16).

Finally, pre-nCRT features were combined with the post-nCRT features to define the delta features as the ratio of the latter to the former, so that a value smaller (bigger) than 1 implies that the post-nCRT feature value has decreased (increased) with respect to the pre-nCRT value.

### Statistical Analysis

For radiomics features analysis, the dataset was randomly split into 90% training and cross-validation data and 10% testing data. The time of distant metastases (DMs) was calculated as the difference between the surgery date and the last follow-up date or the date of metastases event. The analyzed outcome was 2yDM rate, defined as the occurrence of DM within 2 years from the date of the surgery.

Features selection was performed using a 5-folds cross-validation method: the training set was divided in 5 combinations of 4 folds, the remaining fold (each unique combination of 20% training data set) was used for cross-validation. For each combination, a univariate analysis using Wilcoxon Mann-Whitney test was performed if the variables did not show a normal distribution, and T-test was used instead if a normal distribution was observed. Features showing statistical significance (p<0.05) at least in three configurations were selected.

Correlation analysis among the selected features was then performed in terms of Pearson correlation coefficient, selecting only those with a correlation inferior to 30%.

The final set of features was used to train 15 different classifiers on the 5-fold partitioned training set, repeating the cross-validation 3 times and using the accuracy as target metric. The up-sampling method was used to handle the outcome class imbalance. The predictive performance of the trained models was then assessed on the testing data and the best performing classifier was chosen maximising the confusion matrix balanced accuracy.

R statistical software version 3.4.4 was used for statistical analysis (R Core Team (2018). R: A language and environment for statistical computing. R Foundation for Statistical Computing, Vienna, Austria. URL https://www.R-project.org/.).

**Figure 2:**
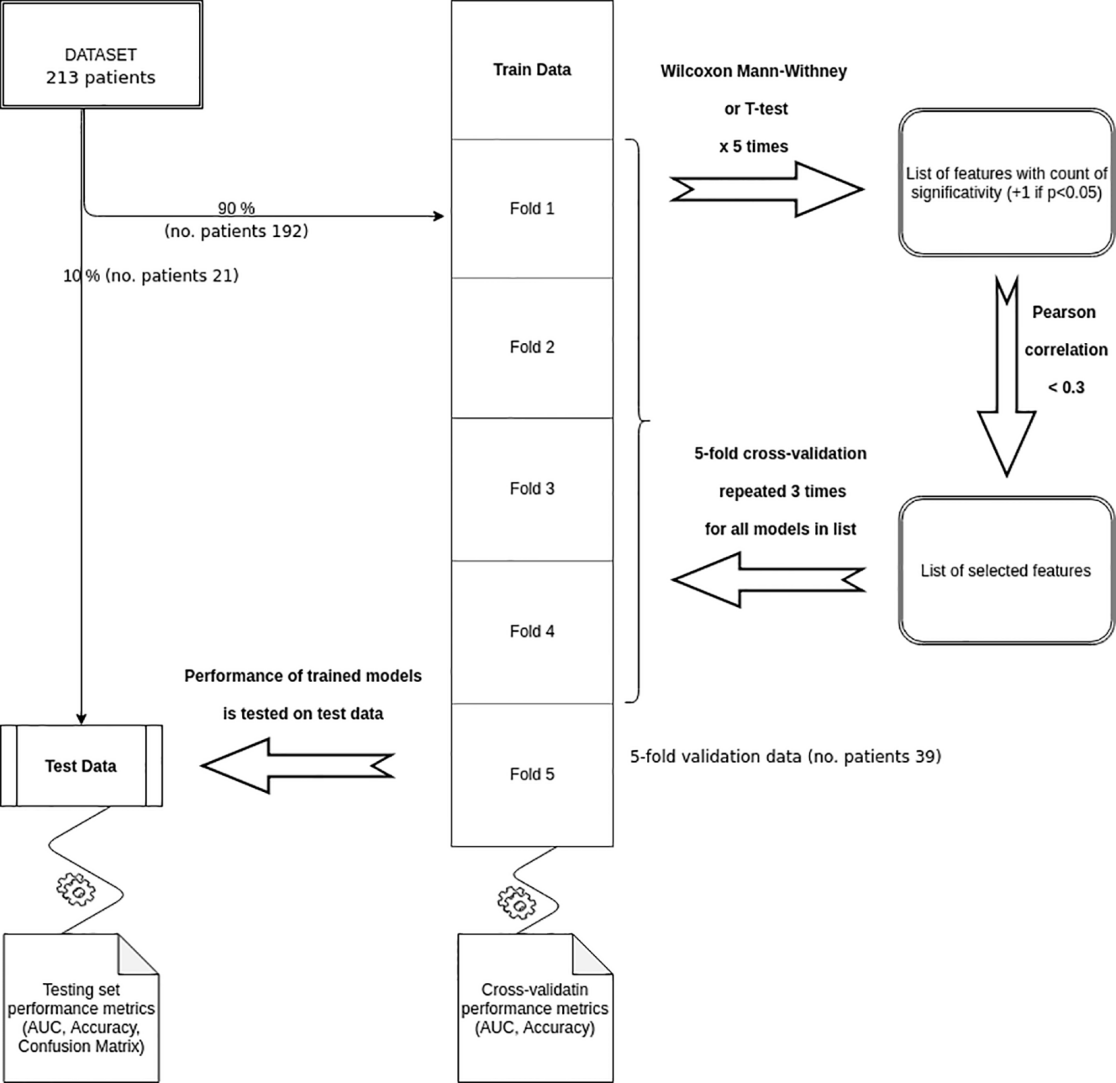
Features selection, models training, and validation processes.

Features selection, models training, and validation processes are shown in **Figure 2**.

## Results

From May 2008 to June 2015 out of 580 LARC patients consecutively treated, 213 patients (37%) met enrolment criteria. In fact, 186 patients (32%) were excluded because the staging and/or restaging MRI was performed in another institution; 105 patients (18%) were excluded because they underwent surgery in another institution; 76 patients (13%) had an inadequate follow-up period.

**Figure 3:**
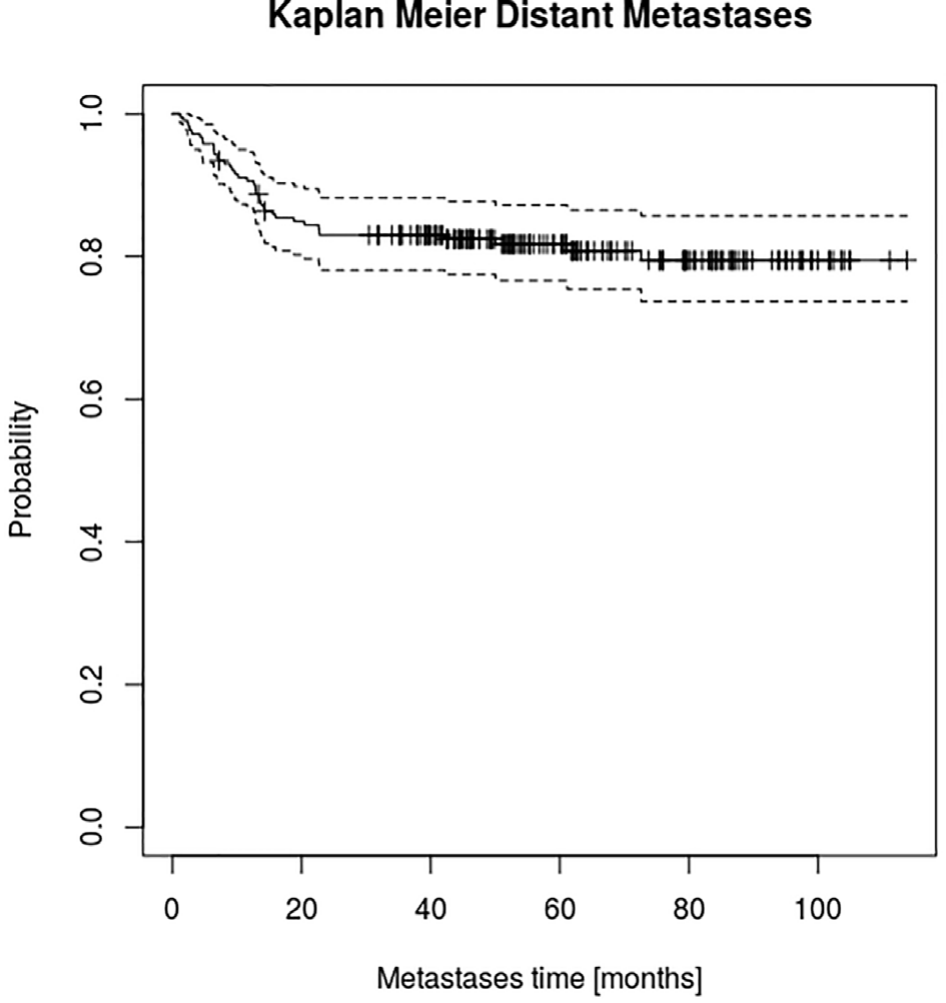
Kaplan-Meier estimator for distant metastasis.

Details of patient characteristics, clinical and treatment features are summarized in **Table 1**. At a median follow-up of 61 (14–119) months, the 2yDM rate was 17% (**Figure 3**) and the median OS of 65 months (13.7–118.8).

**Table 1:** Patient characteristics, clinical and treatment features.

	Patient characteristics and clinical features			
Patient number	213			
	Overall dataset	Training set	Validation set	P-value
Median age at diagnosis [years] (range)	64 (26-83)	64 (28 - 83)	57 (26 - 79)	0.31
Median interval between end of nCRT and surgery [weeks] (range)	11 (7-24)	10 (7-24)	8 (11-16)	0.65
Median length of nCRT [weeks] (range)	5 (2-9)	5 (2 - 9)	5 (3-8)	0.51
Median time of follow-up [months]	61 (14-119)	60 (13-119)	70 (41-104)	0.23
Median PFS [months] (range)	51 (3-114)	51 (4-114)	49 (3-108)	0.32
Sex				0.60
Male Female	136 (64%) 77 (36%)	121 (63%) 71 (37%)	15 (71%) 6 (29%)	
cT				0.19
2 3 4	14 (7%) 132 (62%) 67 (31%)	11 (6%) 118 (61%) 63 (33%)	3 (14%) 14 (67%) 4 (19%)	
cN				0.35
0 1 2	13 (6%) 68 (32%) 132 (62%)	11 (7%) 59 (30%) 122 (63%)	2 (10%) 9 (43%) 10 (47%)	
ycT				0.42
0 1 2 3 4 NA	36 (17%) 11 (5%) 73 (34%) 70 (33%) 21 (10%) 2 (1%)	29 (16%) 10 (5%) 67 (35%) 64 (33%) 20 (10%) 2 (1%)	7 (34%) 1 (5%) 6 (28%) 6 (28%) 1 (5%) 0 (0%)	
ycN				0.98
0 1 2 3 NA	96 (45%) 86 (40%) 28 (13%) 1 (<1%) 2 (1%)	86 (45%) 78 (40%) 25 (13%) 1 (<1%) 2 (1%)	10 (48%) 8 (38%) 3 (14%) 0 (0%) 0 (0%)	
MRI response type				0.42
Complete Partial Stable	32 (15%) 178 (84%) 3 (1%)	25 (13%) 165 (86%) 2 (1%)	7 (33%) 13 (62%) 1 (5%)	
Radiotherapy Dose				
50.4 Gy 55 Gy	18 (8%) 195 (92%)	15 (8%) 177 (92%)	3 (14%) 18 (86%)	0.43
Concomitant neoadjuvant CT type				0.36
with Oxaliplatinum without Oxaliplatinum	142 (67%) 71 (33%)	127 (66%) 65 (34%)	15 (72%) 6 (28%)	
Adjuvant CT type				0.85
with Oxaliplatinum without Oxaliplatinum no adjuvant CT	76 (19%) 41 (36%) 96 (45%)	69 (36%) 37 (19%) 86 (45%)	7 (33%) 4 (19%) 10 (48%)	
Surgical procedure				0.49
APR AR TEM	48 (23%) 154 (72%) 11 (5%)	43 (22%) 140 (73%) 9 (5%)	5 (24%) 14 (67%) 2 (9%)	
ypT				0.49
0 1 2 3 4 NA	55 (26%) 6 (3%) 60 (28%) 83 (39%) 6 (3%) 3 (1%)	51 (27%) 3 (2%) 53 (28%) 77 (40%) 6 (3%) 2 (1%)	6 (29%) 0 (0%) 7 (33%) 7 (33%) 0 (0%) 1 (5%)	
ypN				0.56
0 1 2 NA	152 (71%) 38 (18%) 8 (4%) 15 (7%)	138 (72%) 35 (18%) 7 (4%) 12 (6%)	14 (67%) 3 (14%) 1 (5%) 3 (14%)	
pCR				0.59
Yes No NA	53 (25%) 156 (73%) 4 (2%)	141 (73%) 48 (25%) 3 (2%)	15 (71%) 5 (24%) 1 (5%)	
Response				0.12
TRG=1 TRG>1 NA	55 (26%) 146 (69%) 12 (5%)	50 (26%) 133 (69%) 9 (5%)	5 (24%) 13 (62%) 3 (14%)	
Distant metastases event at 2 years				0.08
Yes No	36 (17%) 177 (83%)	29 (15%) 163 (85%)	7 (33%) 14 (67%)	

Distant PFS, distant progression-free survival; CT, chemotherapy; pCR, pathological complete response; TRG, tumor regression grade; nCRT, neoadjuvant chemoradiation therapy; AR, anterior resection; APR, abdominal-perineal resection; TEM, transanal endoscopic microsurgery; NA, not available.

Kaplan-Meier estimator for DMs was computed as shown in **Figure 3**.

### Feature and Model Selection

For the delta radiomics analysis, the described feature selection strategy selected 216 features on the first 5-fold run, 390 on the second, 168 on the third, 190 on the fourth, and 275 on the fifth (9, 16, 7, 8, and 11% of the total number of features, respectively). The selected features were 110, equal to 4% of the total. The correlation analysis identified 4 non-collinear features (**Table 2**) which were used to train the 15 classifiers, whose results on the testing set were reported in **Table 3**.

**Table 2:** Final selected features correlation matrix.

	medianFD 30,60.delta	F_szm.lzlge 1.1.delta	F_morph.pca.flatness.pre	F_cm.clust.prom 0.6.pre
medianFD 30,60.delta	1	−0.284	0.090	−0.108
F_szm.lzlge 1.1.delta	−0.284	1	−0.046	−0.022
F_morph.pca.flatness.pre	0.090	−0.046	1	−0.136
F_cm.clust.prom 0.6.pre	−0.108	−0.022	−0.136	1

**Table 3:** Models performance on the testing set taken from the confusion matrix at 0.5 cut-off for probability prediction.

Model	Balanced Accuracy	Accuracy	Specificity	Sensitivity	NPV	PPV	Kappa
**Logistic Regression** (LOGREG)	**0.785**	**0.809**	**0.857**	**0.714**	**0.857**	**0.714**	**0.571**
K-Nearest Neighbors (KKNN)	0.464	0.571	0.785	0.142	0.647	0.250	0.080
Penalized Discriminant Analysis (PDA)	0.464	0.571	0.785	0.142	0.647	0.250	0.080
Shrinkage Discriminant Analysis (SDA)	0.678	0.714	0.785	0.571	0.785	0.571	0.357
High Dimensional Discriminant Analysis (HDDA)	0.607	0.476	0.214	1.000	1.000	0.388	0.153
Nearest Shrunken Centroids (PAM)	0.750	0.761	0.785	0.714	0.846	0.625	0.482
C5.0 Tree (C5TREE)	0.535	0.666	0.928	0.142	0.684	0.500	0.087
Partial Least Squares (PLS)	0.678	0.714	0.785	0.571	0.785	0.571	0.357
Random Forest Default (RF_DEF)	0.642	0.761	1.000	0.285	0.736	1.000	0.347
Random Forest Random Search (RF_RAND)	0.571	0.714	1.000	0.142	0.700	1.000	0.181
Random Forest Grid Search (RF_GRID)	0.571	0.714	1.000	0.142	0.700	1.000	0.181
Support Vector Machine (SVM)	0.607	0.666	0.785	0.428	0.733	0.500	0.222
Naïve Bayes (NB)	0.642	0.571	0.428	0.857	0.857	0.428	0.228
Neural Network (NN)	0.571	0.571	0.571	0.571	0.727	0.400	0.129

The best model was shown in bold.

The highest balanced accuracy model was the logistic regression (0.785) which also had an overall classification performance (specificity: 0.857, sensitivity: 0.714, negative predictive value: 0.857, positive predictive value: 0.714, Cohen’s Kappa statistics: 0.571).

## Discussion

Several studies highlighted the validity of the radiomics approach in rectal cancer, obtaining predictive models that allow identifying responding patients or risk categories for different outcomes using staging MRI (8, 9, 14, 17–23).

Our previous experiences confirmed that the radiomics models based on staging MRI provide a relevant predictive tool to identify tumor behavior in terms of pCR after nCRT (14–16, 24).

Despite the important effort made in terms of treatment response prediction, few experiences reported the relation between radiomics predictors and early distant recurrence (14–16).

In the framework of personalized medicine, a new radiomics approach is spreading in the scientific literature, called delta radiomics, which aims to elaborate predictive models analysing the variation of radiomics features extracted from images acquired before and after the treatment and including information regarding the response to the treatment of the individual patient.

In this context, some experiences tried to predict tumor behavior considering clinical features (4) or merged texture analysis features in addition with morphological MRI and histopathological parameters for both staging and post-nCRT MRI (21) or just on the staging MRI (25).

Liu et al. (26), investigated the predictive role of pre-nCRT MRI radiomics parameters to predict synchronous DM in 177 rectal cancer patients with an area under the curve of receiver operating characteristic of 0.827.

Liang et al. (31) analyzed the differences between metastatic and non-metastatic patients using a support vector machine and identifying MRI radiomics features able to predict metachronous liver metastasis in a cohort of 108 patients with an AUC of 0.87.

Jeon et al. (32). identified a nomogram to predict, using delta-radiomics signatures, LR, DM and DFS on 101 patients (67 patients for model training, 34 for internal validation).

Our study was focused on the 2yDM prediction in patients affected by LARC based on a larger retrospective cohort than the one reported by Jeon et al.

Starting from clinical nomogram based on a pooled analysis (27) ypN stage, ypT stage, surgery procedure and adjuvant chemotherapy (CT) seem to contribute to DM prediction. Furthermore, the role of adjuvant CT is still controversial with only small benefit in high-risk group (33, 34).

The value of a stronger predictive model of early systemic disease in LARC patients could help to identify the subset of patients with a higher risk of DM for a tailoring specific adjuvant treatment.

This personalized approach may allow avoiding unnecessary systemic toxicities for patients with low risk of DM, considering the small contribute of CT in this subset of patients.

On the other hand, treatment intensification, based on a multidrug combination or personalized approaches, could be designed for patients with a high risk of early development of DM.

Using a delta radiomics approach, with a logistic regression classifier, we built a model with a balanced accuracy, accuracy, specificity and sensitivity of 0.785, 0.809, 0.857, and 0.714, respectively.

There are several limitations in this study: first, the lack of an external validation with an independent dataset of patients, mandatory to confirm the applicability of the model in a cohort of patients from other institutions (35). The known variability in MRI acquisition parameters and the signal obtained from different patients, scanners and protocols, pose an additional challenge to the reproducibility of radiomic signatures and represent sources of uncertainty. In fact, despite for this study all MRI were acquired using the same protocol and the same MRI scanner, the applicability of this model is tightly linked to the opportunity to conduct an external validation with an independent dataset to confirm the MRI vendor-independency of delta-radiomics features, as previous confirmed for radiomics ones (15). Other limitations of our study are the lack of other prognostic, clinical, histological and genetic endpoints in the analysis, which would allow to perform a multivariate analysis and build a more robust hybrid predictive model.

Despite the disclosed limitations, this paper shows the relevance of the delta radiomics approach to predict the subset of patients with a higher risk of 2yDM in a large single-institution cohort.

In conclusion, delta radiomics is a promising imaging biomarker that can estimate the disease’s behavior in LARC, predicting the risk of early systemic recurrence. Early diagnosis of aggressive tumors may represent a significant added value in order to offer innovative personalized and tailored treatments, allowing physicians to guide their choices avoiding unjustified toxicity or preferring an intensified treatment when necessary.

## Data Availability Statement

The raw data supporting the conclusions of this article will be made available by the authors, without undue reservation.

## Author Contributions

GC, PR-C, JL, CC, CM, DCu, DCa, and LB participated in developing the concept of this manuscript, imaging segmentation, data analysis, researching and writing, manuscript preparation, and approval of the final manuscript draft. ND, EM, AD, BB, RM, VV, and MG participated in researching and writing this manuscript, manuscript preparation, and approval of the final manuscript draft. All authors contributed to the article and approved the submitted version.

## Funding

The participation of one of the authors was supported by an ESOR-Bracco Research Grant.

## Conflict of Interest

The authors declare that the research was conducted in the absence of any commercial or financial relationships that could be construed as a potential conflict of interest.
